# Long-term effectiveness and drug survival of golimumab in patients affected by psoriatic arthritis with cutaneous involvement

**DOI:** 10.1007/s10067-021-05874-6

**Published:** 2021-08-19

**Authors:** Maria Sole Chimenti, Paola Conigliaro, Francesco Caso, Luisa Costa, Augusta Ortolan, Paola Triggianese, Marco Tasso, Giulia Lavinia Fonti, Maria Grazia Lorenzin, Roberto Perricone, Roberta Ramonda

**Affiliations:** 1 Rheumatology, Allergology and Clinical Immunology, Department of Medicina Dei Sistemi, University of Rome Tor Vergata, Via Montpellier 1, Rome, Italy; 2 Rheumatology Unit, Department of Clinical Medicine and Surgery, University Federico II, Naples, Italy; 3 Rheumatology Unit, Department of Medicine DIMED, University of Padova, Via Giustiniani 2, Padua, Italy

**Keywords:** Drug survival, Golimumab, Long-term effectiveness, Psoriasis, Psoriatic arthritis, Real-life

## Abstract

**Objectives:**

To determine the effectiveness of golimumab (GLM) in improving joint, periarticular structures and cutaneous manifestations in patients with moderate to severe psoriatic arthritis (PsA) with cutaneous psoriasis in different real-life clinical settings and 48-month drug survival.

**Methods:**

Clinical and laboratory records were collected from PsA patients treated with GLM at baseline (T0) and after 6, 12, 24, 36, and 48 months of treatment. Comparisons were performed using a paired *t*-test or Wilcoxon test. Drug survival rates were analyzed using Kaplan–Meier estimates. *p* value < 0.05 was considered statistically significant.

**Results:**

Data from 105 patients were collected. PsO occurred in 80% of patients and enthesitis in 78%, peripheral and axial arthritis in 63.8% and 35.3%, respectively, while erosions in 36.2%. The main comorbidities were cardiovascular diseases (31.4%) and metabolic syndrome (MetS) (19%). A statistically significant improvement in articular and cutaneous psoriasis was registered at T48 of GLM-therapy in clinical (DAPSA *p* < 0.0001; PASI *p* < 0.01; BASDAI *p* < 0.0001) and laboratory (CRP < 0.05) indexes. Gender (*p* = 0.652), BMI (*p* = 0.655), smoking habit (*p* = 0.466), and line of treatment (*p* = 0.208) did not affect treatment efficacy nor persistence. At T48, 42% of patients discontinued GLM: the most frequent reason was an insufficient response or loss of efficacy (28.6%).

**Conclusion:**

A 48-month GLM high drug persistence of PsA patients was observed in real-life, in patients presenting high disease activity, elevated prevalence of comorbidities, and more than one line of treatment at baseline. Patients’ characteristics as gender, smoke, BMI, different lines of treatment, and concomitant methotrexate treatment affected treatment persistence, making GLM effective and safe in moderate-severe PsA in a long-term real-life setting.

**Table:** 

**Key Points** • *Golimumab was effective in psoriatic arthritis, including both musculoskeletal and cutaneous manifestations. * • *Golimumab effectiveness and drug survival were not affected by comorbidities and patient-related characteristics. * • *The 4-year drug survival curves confirm the efficacy and safety of golimumab in psoriatic arthritis patients in a real-life setting. *

## Introduction

Psoriatic arthritis (PsA) is a chronic inflammatory disease, belonging to the group of spondyloarthritis (SpA), typically associated with psoriasis (PsO) and characterized by the presence of both articular and periarticular structures involvement [1]. The prevalence of PsO ranges from 2 to 3% in the general population, and PsA may affect 10–30% of PsO patients [2]. Joint involvement in PsA is a potentially debilitating disease: patients may present arthritis to both small and large peripheral joints, enthesitis, and the axial disease, leading to progressive erosive arthritis and severe functional impairment in more than half of the patients [3]. PsA pathogenesis is only partially understood and still remains to be completely clarified. The presence and the overexpression of cytokines such as tumor necrosis factor (TNF)-α, interleukin (IL)-17, and IL-23, plays a role in the pathogenic ways linking PsA and PsO [4]. The use of TNF inhibitors (TNFi) approximately two decades ago has dramatically improved PsA treatment. However, several unmet needs concerning the management of both joint and skin manifestations still remained [5]. Randomized-placebo controlled clinical trials involving TNFi have shown excellent results for PsA and PsO treatment. Etanercept, infliximab, adalimumab, golimumab (GLM), and certolizumab have been demonstrated to be effective in PsA and in distinctive aspects of the disease, as skin disease, peripheral arthritis, enthesitis, and dactylitis. Moreover, their effectiveness was demonstrated in improving quality of life and work ability and in reducing radiographic progression [6]. GLM is a TNFi approved by the European Medicines Agency (EMA) and the US Food and Drug Administration (FDA) for the treatment of active rheumatoid arthritis, active PsA, active ankylosing spondylitis, severe non-radiographic axial SpA, polyarticular juvenile idiopathic arthritis, and moderate to severe active ulcerative colitis [7]. GLM is a fully human monoclonal antibody IgG1k-neutralizing TNF-α. Clinical trials as GO-REVEAL studies demonstrated a satisfactory efficacy of GLM in improving PsA signs and symptoms and in treating the structural damage caused by the disease [8]. Treatment recommendations underline the relevance of the burden of skin PsO in patients affected by PsA and skin manifestations need to be a relevant part of the decision-making process for PsA therapeutic approach [9]. However, the effectiveness evaluation of GLM in a real-life setting is a crucial issue, in particular for skin manifestation. To date, only a few unsponsored long-term studies reported the efficacy of GLM treatment in PsA patients and their treatment adherence and, to our knowledge, the GLM effectiveness according to patients characteristics and lines of treatment (i.e., 1st, 2nd, and 3rd or more lines) was rarely considered [10, 11]. Moreover, limited data are available on the effectiveness and safety of GLM in patients affected by both PsO and PsA in bio-naïve and TNF-insufficient responders (TNF-IR), in particular concerning skin sustained efficacy.

To address these knowledge gaps, the aims of this multicenter observational study were to evaluate the efficacy and the long-term treatment retention rate in patients affected by PsA with associated PsO treated with GLM in a real-life setting. Moreover, we aimed at evaluating the treatment retention rate based on patient characteristics, such as gender, body mass index (BMI), presence of comorbidities, smoking habit, and line of GLM treatment.

## Materials and methods

### Patients

A retrospective observational study was conducted on consecutive outpatients diagnosed with moderate-severe PsA treated with GLM between August 2011 and December 2020 attending the combined derma-rheuma clinic in the Rheumatology Units of the University of Padova, the University of Rome Tor Vergata, and the University of Naples Federico II. The end-points of the study were to determine efficacy on both joint and skin manifestations in PsA patients, the retention rate, and safety profile of a 4-year follow-up of GLM treatment. Inclusion criteria were the following: (1) age > 18 years; (2) diagnosis of PsA > 6 months in accordance with the Classification for Psoriatic Arthritis (CASPAR) criteria [12] and concomitant PsO; (3) the occurrence of peripheral arthritis (at least 1 active joint) and active PsO according to Psoriasis Area Severity Index (PASI) score [13]; (4) indication to start GLM treatment.

Data were collected at baseline (T0) and after 6 (T6), 12 (T12), 24 (T24), and 48 (T48) months of GLM treatment. PsO was clinically diagnosed by an experienced dermatologist (MT) and inflammatory bowel disease (IBD) was assessed as active or inactive based on the gastroenterologist (EC) (clinical and/or endoscopic) evaluation. Patients’ comorbidities were evaluated in accordance with the classification of diseases outlined in the Charlson Comorbidity Index [14]. The presence of comorbidities and concomitant therapies were investigated (yes/no) at all the scheduled assessments by reviewing the patients’ medical records. Metabolic syndrome (MetS) was investigated in accordance with internationally recognized standards [14, 15]. Previous therapies with biologic disease-modifying antirheumatic drugs (bDMARDs), use of concomitant conventional synthetic DMARDs (csDMARDs) (methotrexate-MTX, leflunomide, sulfasalazine, and cyclosporine A), non-steroidal anti-inflammatory drugs (NSAIDs), and corticosteroid therapy were registered at baseline and during the follow-up.

The comprehensive evaluation by clinical examination, made by experienced rheumatologists (MSC, FC and AO), included Bath Ankylosing Spondylitis disease activity index (BASDAI), Ankylosing Spondylitis Disease Activity Score-C-reactive protein based (ASDAS-CRP), evaluation of dactylitis (yes/no), enthesitis (yes/no), tender joint count (in 68 joints), swollen joint count (in 66 joints), and Disease Activity in Psoriatic Arthritis (DAPSA) score. Both the PASI and the assessment of psoriatic onychopathy (yes/no) were made by an experienced dermatologist. Patients were evaluated for the occurrence of erosions in peripheral joints by radiographic exams by three experienced rheumatologists (PC, MGL, and MT). Axial involvement was defined as the presence of radiographic axial involvement (at least unilateral grade 2 sacroiliitis and/or either marginal or paramarginal syndesmophytes in the cervical, thoracic, or lumbar spine on spinal radiographs) or by the presence of MRI sacroileitis [16]. Patient-reported outcomes (PROs) comprised pain visual analogue scale (pVAS) and global health (GH) and health assessment questionnaire modified for spondyloarthritis (HAQ-S). Inflammatory markers as erythrocyte sedimentation rate (ESR) and CRP were registered. Metrological indexes such as height, weight, BMI, and abdominal circumference were evaluated. All the patients were treated with subcutaneously GLM at a dosage of 50 mg or 100 mg depending on the weight (100 mg was used when the patient’s weight was ≥ 100 kg) at week 0 and every 4 weeks thereafter, in accordance with the manufacturers’ instructions [7].

The drug’s safety was evaluated by assessing adverse events and via standard laboratory testing, the retention rate was estimated at 6, 12, 24, and 48 months of treatment.

The study was approved by the local ethics committees of the institutions involved. Informed consent was obtained from all the patients before they were included in the study that was conducted in accordance with the ethical principles of the Declaration of Helsinki and consistent with good clinical practice guidelines.

### Statistical analysis

To test the normality of data sets, the D’ Agostino and Pearson omnibus test was used. Continuous data are presented as mean and standard deviation (SD) or median and interquartile range (IQR) when appropriate. For categorical variables, absolute and relative frequencies are reported. Continuous variables were compared using a paired *t*-test or a Wilcoxon signed-rank test for paired samples. Categorical variables were compared using the chi-squared test or Fisher’s exact test when appropriate. The drug survival rates were analyzed using the Kaplan–Meier estimates. Drug survival rates were read from the Kaplan–Meier survival curves. Differences in drug survival between groups were analyzed using a log-rank (Mantel-Cox) test, by stratifying for sex, BMI, smoking habit, and line of treatment. A *p* value < 0.05 was considered statistically significant. All statistical analyses were performed using GraphPad Prism version 7 (GraphPad software).

## Results

### Patient characteristics

Data from a total of 105 patients affected by moderate to severe PsA who started treatment with GLM were registered. Baseline clinical and laboratory characteristics are summarized in Table 1.

**Table 1:** Baseline clinical and serological characteristics of patients

*N*	105
Age, years	53.04 ± 11.6
M/F	44/61
Disease duration, months	174.9 ± 93.0
PsO, *n* (%)	105 (100%)
Peripheral disease, *n* (%)	68 (64.7%)
Tender joint count	8.1 ± 5.5
Swollen joint count	2.7 ± 3.1
Axial disease, *n* (%)	37 (35.3%)
Enthesitis, *n* (%)	82 (78%)
Dactylitis, *n* (%)	36 (34.2%)
ESR, mm/h	24.1 ± 18.9
CRP, mg/dL	1.2 ± 2.3
DAPSA	25.7 ± 9.7
BASDAI	6.8 ± 2.5
PASI	2.3 ± 3.5
ASDAS-CRP	3.3 ± 0.8
Active IBD Crohn disease, *n* (%) RCU disease, *n* (%)	7 (6.6%) 2 (1.9%) 5 (4.8%)
Inactive IBD, *n* (%)	13 (12.4%)
Inactive uveitis, *n* (%)	1 (0.9%)
BMI	27.1 ± 6.0
HLA-B27 positive, *n* (%)	11 (10.5%)
Smoking, *n* (%)	33 (31.4%)
Cardiovascular comorbidities, *n* (%)	33 (31.4%)
Metabolic comorbidities, *n* (%)	20 (19%)
Hashimoto’s disease, *n* (%)	7 (6.7%)
Concomitant csDMARD, *n* (%)	60 (57.1%)
Concomitant glucocorticoids, *n* (%)	40 (38.1%)
Concomitant NSAIDs, *n* (%)	42 (40%)
Biologic-naïve, *n* (%)	44 (41.9%)
GLM as second line, *n* (%)	25 (23.8%)
GLM as third line, *n* (%)	27 (25.7%)
GLM as fourth line or more, *n* (%)	9 (8.6%)

Data are expressed as mean ± standard deviation

*M* male, *F* female, *PsO* psoriasis, *GLM* golimumab, *NSAIDs* non-steroidal anti-inflammatory drugs, *BMI* body mass index, *csDMARD* conventional synthetic disease-modifying antirheumatic drugs, *ESR* erythrocyte sedimentation rate, *CRP* C-reactive protein, *DAPSA* disease activity in psoriatic arthritis, *BASDAI* Bath Ankylosing Spondylitis disease activity index, *ASDAS-CRP*, Ankylosing Spondylitis Disease Activity Score-C-reactive protein based, *IBD* inflammatory bowel diseases, *PASI* Psoriasis Area Severity Index

Peripheral arthritis was present in 68 (64.8%) cases and axial disease in 37 cases (35.2%). In particular, oligoarthritis was denoted in 30 cases (28.6%) and polyarthritis in 75 cases (71.4%). In the group of patients with axial disease, single-joint arthritis occurred in 17 patients (45.9%), oligoarthritis in 6 patients (16.2%), and polyarthritis in 10 cases (27%). No cases of arthritis *mutilans* or prominent distal interphalangeal joint (DIP) involvement were registered. Enthesitis and PsO were described in 82 (78%) and 105 (100%) patients, respectively. Moreover, peri-articular manifestations were reported in 36 (34.2%) patients. Extra-articular manifestations including IBD were observed in 20 (19%) patients (13 with inactive IBD and 7 patients with active disease) while isolated anterior uveitis occurred in a single case (0.9%).

The most frequent comorbidity described was the occurrence of cardiovascular diseases in 33 (31.4%) patients and MetS in 20 (19%) patients. A relevant incidence of smokers (33 patients, 31.4%) was registered as well as an elevated mean BMI score (27.1 ± 6.0), with overweight in 39 (37.1%) cases and a defined obesity in 20 (19%) cases.

### Golimumab effectiveness

**Fig. 1:**
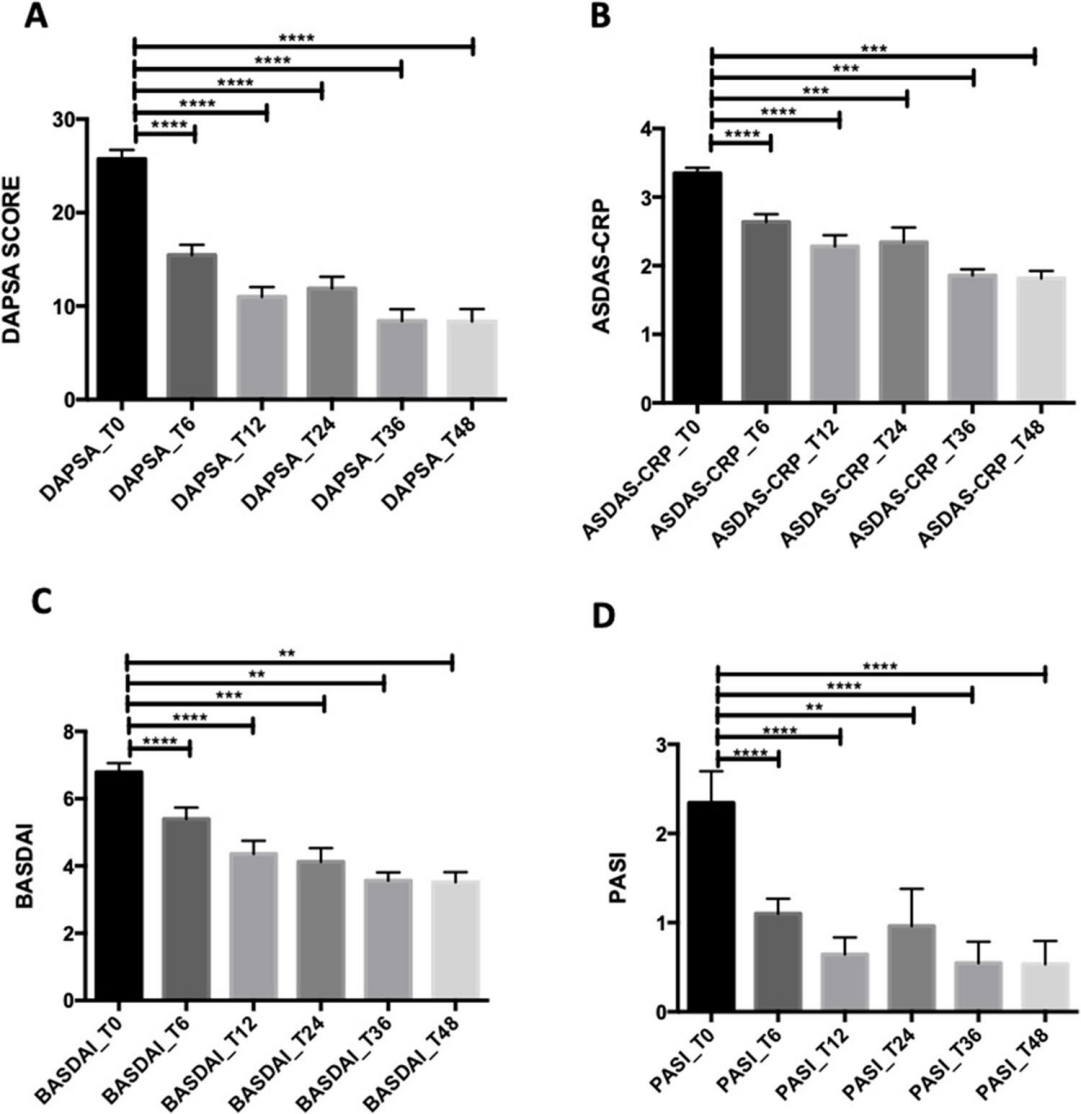
**A–D** Golimumab effectiveness measured by composite clinimetric indexes and PASI score. **p < 0.01; ***p < 0.001; ****p < 0.0001. *DAPSA* Disease Activity in Psoriatic Arthritis, *BASDAI* Bath Ankylosing Spondylitis disease activity index, *ASDAS-CRP* Ankylosing Spondylitis Disease Activity Score-C-reactive protein based, *PASI* Psoriasis Area Severity Index

According to the primary end-point of the study, a statistical significant improvement of the DAPSA score was observed at T6, T12, T24, T36, and T48 months (*p* < 0.0001 for all comparisons, Fig. 1A). ASDAS-CRP levels significantly reduced at all time points (*p* < 0.0001 at T6, *p* < 0.0001 at T12, *p* = 0.0001 at T24, *p* = 0.0005 at T36, and *p* = 0.0002 at T48, Fig. 1B) while BASDAI score reduced at T6 (*p* < 0.0001), T12 (*p* < 0.0001), T24 (*p* = 0.0002), T36 (*p* = 0.004), and T48 (*p* = 0.002) (Fig. 1C). A significantly reduction in the frequency of dactylitis was observed at T6 (*n* = 9, 8.6%), T12 (*n* = 2, 1.9%), T24 (*n* = 2, 1.9%), T36 (*n* = 0, 0%), and T48 (*n* = 0, 0%). This data was also confirmed for enthesitis at T6 (*n* = 52, 49.5%), T12 (*n* = 16, 15.2%), T24 (*n* = 7, 6.6%), T36 (*n* = 2, 1.9%), and T48 (*n* = 2, 1.9%). A substantial improvement was also detected in skin involvement: PASI score at T6 (*p* < 0.0001), T12 (*p* < 0.0001), T24 (*p* = 0.001), and T36 and T48 (*p* < 0.0001 for both comparisons) (Fig. 1D).

**Fig. 2:**
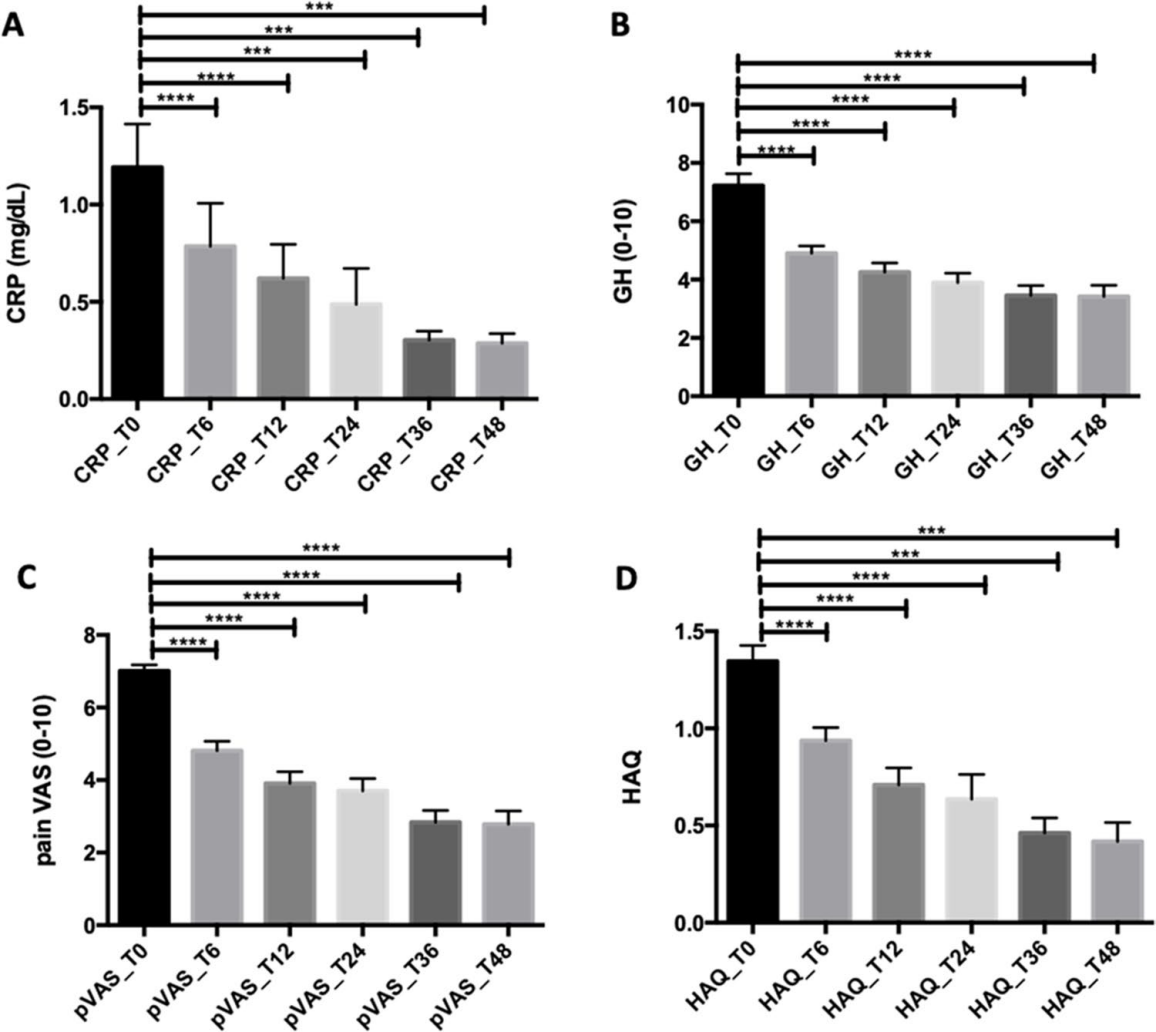
**A–D** Golimumab effectiveness measured by patient-reported outcomes and inflammatory markers. **p < 0.01; ***p < 0.001; ****p < 0.0001. *CRP* C-reactive protein, *GH* global health, *pVAS* pain-visual analogue scale, *HAQ* health assessment questionnaire

CRP levels decreased at T6, T12, T24 (*p* < 0.0001 for all comparisons); T36 (*p* = 0.0009); and T48 (*p* = 0.0007) (Fig. 2A). GH and pain were reduced at all the time points (*p* < 0.0001 for all comparisons), and disability improved at T6 (*p* < 0.0001), T12 (*p* < 0.0001), T24 (*p* < 0.0001), T36 (*p* = 0.0005), and T48 (*p* = 0.0002) (Fig. 2B–D). Clinical improvement was also evaluated by the gain of DAPSA low disease activity: at T6 (*n* = 54) 62.1%, T12 (*n* = 49) 63.6%, T24 (*n* = 36) 55.4%, T36 (*n* = 35, 61.5%), and T48 (*n* = 35, 61.5%) and DAPSA remission score: at T6 (*n* = 11) 12.6%, T12 (*n* = 16) 20.8%, T24 (*n* = 17) 26.2%, T36 (*n* = 21) 36.8%, and T48 (*n* = 21) 36.8%.

### Drug survival and safety

In order to explore the clinical response, survival rate curves at 6, 12, 24, and 48 months, as well as the reasons for treatment discontinuation, were assessed.

**Fig. 3:**
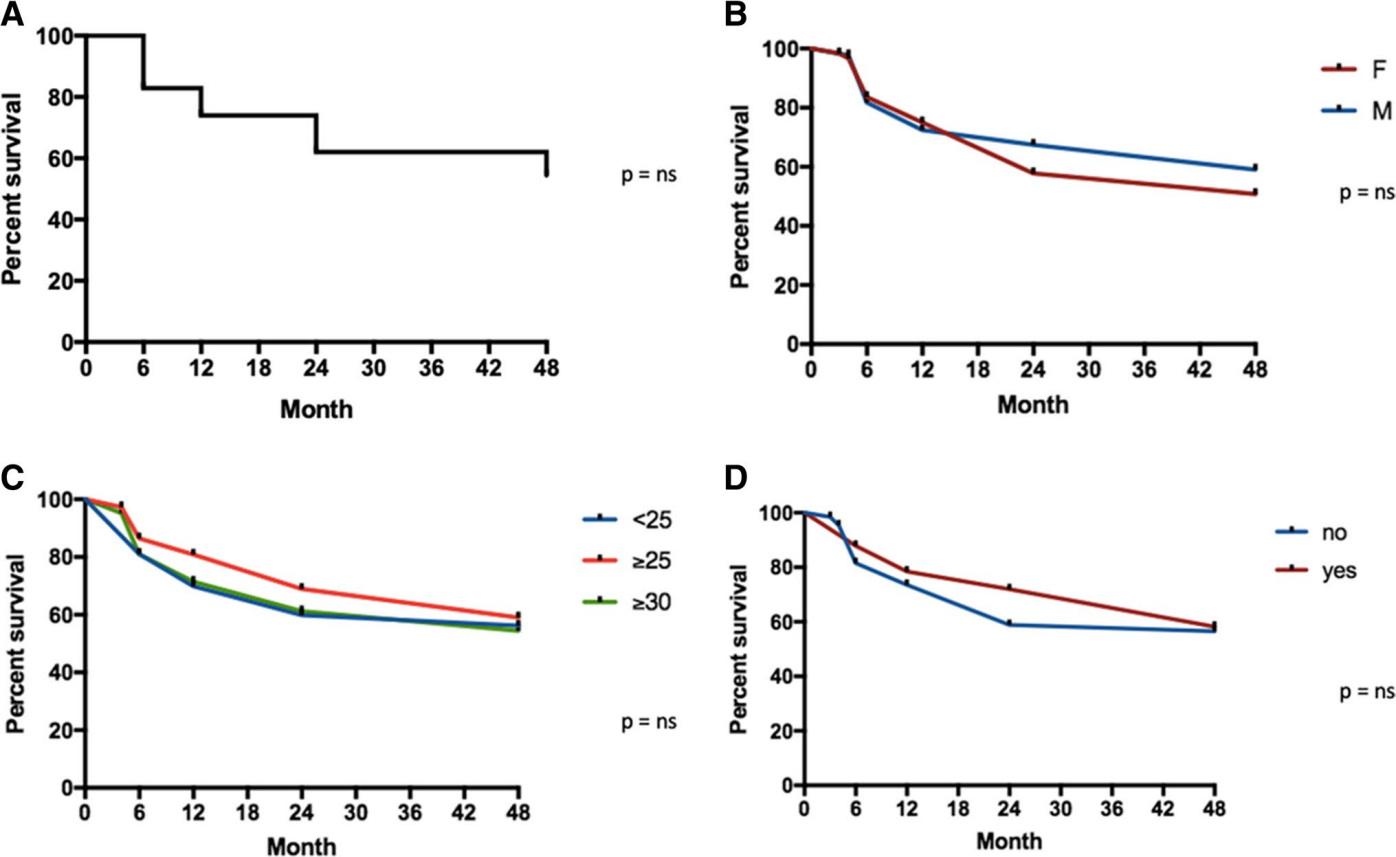
**A**–**D**. Forty-eight-month retention rate global (**A**) and according to gender (**B**), BMI (**C**), and smoke habit (**D**). *M* male, *F* female; < 25 normal weight, > 25 overweight, > 30 obese

**Fig. 4:**
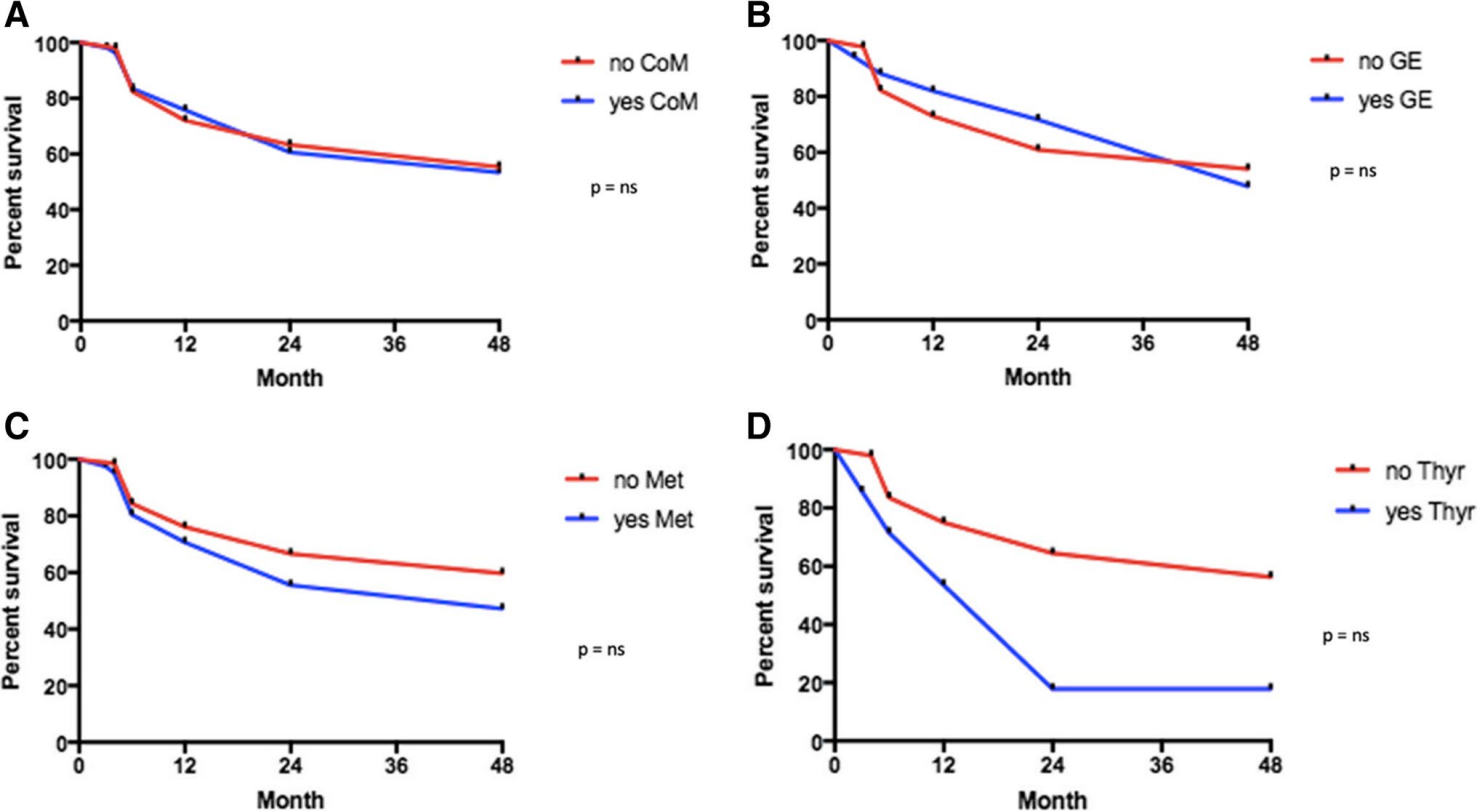
**A–D** Forty-eight-month retention rate according to comorbidities (**A**), gastro-enteric pathologies (**B**), metabolic dysfunctions (**C**), and thyroid disease (**D**). *CoM* comorbidities, *GE* gastro-enteric, *Met* metabolic dysfunctions, *Thyr* thyroid disease

Figure 3A shows the GLM retention rate estimated by the Kaplan–Meier analysis. After 6 months of treatment, the retention rate was 82.8%, at 12 months 73.4%, at 24 months 62%, and at 48 months 54.4%. No statistically significant difference emerged when patients were divided according to gender (*p* = 0.652) (Fig. 3B). Similarly, the division of patients into 3 categories based on their BMI (normal weight with BMI < 25, overweight with BMI ≥ 25, and obesity with BMI ≥ 30) did not conduce to any statistically significant result (*p* = 0.655) (Fig. 3C). Data were analyzed by distinguishing patients based on smoking habits and the occurrence of comorbidities. In relation to smoking, no difference was found between smokers and non-smokers (*p* = 0.466) (Fig. 3D). With regard to the presence of comorbidities, no differences were noted between groups (*p* = 0.902) (Fig. 4A). On the other hand, the analysis of the three distinct subgroups of comorbidities highlighted that the presence of a thyroid disease condition was a negative prognostic factor in treatment persistence (*p* = 0.030) (Fig. 4B–D), while no statistically significant correlation was observed between the presence of MetS and persistence in therapy (*p* = 0.238) and between gastro-enteric pathologies and persistence in therapy (*p* = 0.867).

Finally, data were analyzed by focusing on lines of treatment and association with MTX. Concerning lines of treatment, patients were divided into biological naïve and patients who had previously taken other biologicals: there were no statistically significant differences between these two study populations (*p* = 0.208). Based on the association or not with MTX, considering valid the combination therapy only if carried out for a period of time equal to or greater than half of the total duration of follow-up: no difference was found in patients treated as monotherapy compared with those taking MTX (*p* = 0.599).

Overall, in the follow-up, reasons for drug discontinuation were primary inefficacy in 13 cases, secondary inefficacy in 21 cases, and 5 cases were lost at follow-up. For the event, reasons for discontinuation were mild infections in 5 cases and surgery in 1 case not related to the treatment.

## Discussion

In this observational retrospective, multicenter study in patients with moderate to severe PsA with cutaneous manifestations of PsO, GLM was effective for both articular and skin involvement. Indeed, GLM was effective in reducing all clinical parameters analyzed at all the time points of evaluation until 48 months of observation including inflammatory indexes and pain. Data at 36 months were not shown because stackable with those at 48 months. Clinical phenotype of patients was characterized by cutaneous involvement represented in all patients while, concerning articular and peri-articular manifestations, a high prevalence of polyarticular involvement and enthesitis was found. Long-standing disease, high prevalence of erosions, and presence of axial disease demonstrated the presence of a challenging study population of PsA patients. Moreover, the high disease activity at baseline measured by specific composite scores supported the severity of PsA. The statistically significant reduction of clinimetrics indexes, inflammatory markers, and the gain of a high percentage of patients in DAPSA low disease activity support the efficacy of GLM in our “difficult-to-treat” PsA patients. Remission according to DASPA score, an ambitious endpoint, was obtained in 20% of patients at T12 and 36.8% of patients were in clinical remission at T48. In this context, we assume that TNF inhibition represents one of the key treatments for PsA and PsO, as confirmed by clinical trials demonstrating the efficacy of TNFi in both PsA and PsO [5]. The reduction of inflammatory markers, such as CRP, considered a biomarker of radiographic progression in SpA, may be considered as an indirect outcome of the reduction of radiographic damage [17]. Based on ASDAS-CPR and BASDAI scores, significant improvement was reached and maintained within the first 12 months of treatment and sustained during the following 48 months of follow-up for axial involvement. Most patients evaluated in our study had been previously treated with other biologic agents (TNFi) and only 41.9% of patients were bio-naïve with 57% in concomitant csDMARD treatment. The drug survival of GLM was independent of the line of treatment used and the previous use of bDMARDs and can be considered effective both as first-line therapy and in patients with failure for one or more bDMARDs. Data from international and national registries, such as the GISEA registry or the BioRx.si, have compared the characteristics of patients starting treatment with GLM [11, 18, 19]. bDMARD-naïve and bDMARD-experienced patients were compared and no difference in the persistence of GLM was found. The switch among the same group of drugs, as the TNFi family, is still a debate in PsA management and patients failing a first TNFi agent usually use a different target [11]. However, several data from the literature seem to support the efficacy of switching among TNFi in PsA [10, 19]. The evaluation of drug persistence in our real-life experience demonstrated a good percentage (54%) of patients still on therapy after 4 years of GLM treatment. Other studies, evaluating the same outcome, estimated approximately 47% of PsA patients, from the UK, remained on their initial TNFi therapy after 5 years and low persistence of 32% after 5 years of follow-up on PsA patients in Germany, while no differences among TNFi were observed [20–22].

Recently, none of the TNFi agents was found to be more persistent than others as first-line therapy, while secukinumab was found to be superior to other biologics when indicated as second-line therapy [23]. However, data from Slovenia showed a better persistence of GLM compared to other TNFis in bDMARD-experienced AS and PsA patients [19].

The use of combination therapy as bDMARDs with MTX or other csDMARDs is still controversial in the management of PsA since its rational use is based on indirect evidence or expert opinion [24]. However, csDMARDs may be warranted in peripheral joint manifestations when bDMARDs are not effective [25]. We have not observed significant differences in survival rates among patients using MTX or not, confirming that GLM was effective in both combination treatment and monotherapy. This result suggests to using the GLM also in those patients that are intolerant to csDMARDs, making MTX treatment not necessary when a bDMARD is started. Furthermore, no difference in survival rates was observed when patients were divided according to the presence of comorbidities, BMI, or gender.

MetS was strongly associated with a lower probability to achieve MDA in TNFi-treated PsA patients, and different studies reported a higher prevalence of MetS in PsA patients compared to other rheumatic diseases [26, 27]. In our cohort of patients, MetS was not a negative predictor factor for GLM efficacy, in contrast with other findings supporting the hypothesis that MetS or overweight and obesity may affect TNFi therapy, as adalimumab or etanercept. GLM efficacy might not be affected by the presence of MetS because the dosing regimen is adequate to the weight of the patients being MetS related to chronic inflammatory state and adipokines. Lifestyle such as smoking habit is associated with high disease activity in PsA patients and may exert a negative role during the treatment with bDMARDs [28]. We have not observed the influence of smoking in our 4 years of follow-up, making GLM effective also in smokers.

Among other factors affecting treatment response, gender may influence bDMARDs clinical response remaining a challenge in PsA. In particular, female gender has been associated with a prevalent polyarticular phenotype, high swollen joint count, and peripheral erosive joint involvement compared to males, while female gender correlated with lower rates of extraarticular manifestations and axial involvement [28, 29]. Moreover, the female gender generally associates with poor rates of response to TNFi and a lower probability of achieving remission compared to males [30], especially when comorbidities, such as MetS, occur [30–32]. In our cohort, we did not observe gender differences in the efficacy of GLM, since gender was not a relevant factor in patient outcomes. The high prevalence of IBD in our cohort is due to the early referral in the combined gastro-rheuma outpatient clinic by gastroenterologists from the involved hospitals [33, 34]. Thyroid dysfunction may be associated with the worst clinical outcome in several inflammatory conditions and in PsA [26, 35, 36]. According to our data, thyroid disorders may represent a negative prognostic factor in the PsA study population for treatment persistence even if this result needs to be confirmed in a larger study cohort. Thyroid disorders may have a relevant impact on disease perception and activity and so may have an influence on treatment efficacy.

A very low rate of primary and secondary failure was observed with a good safety profile with no serious adverse events reported consistent with previous reports of patients treated with GLM [37–39].

This study encompasses limitations such as the retrospective nature of the study, the presence of low cutaneous disease activity at baseline, the relatively low number of PsA patients enrolled due to the inclusion of patients with both joint and skin manifestations, and the absence of imaging in the follow-up (radiographic evaluation of efficacy), even if ultrasound, MRI, and/or X-ray was performed in all patients to confirm the diagnosis at baseline.

## Conclusion

In this multicentric study, we described the clinical efficacy in joint and periarticular structures as well as on PsO manifestations on moderate to severe PsA patients treated with GLM in a real-life setting. Treatment efficacy was supported by a good drug persistence of GLM regardless of the presence of comorbidities and patients’ characteristics. Our results were consistent with no differences in terms of clinical response and efficacy between males and females, smokers and no-smokers, obese and normal-weight patients, and lines of treatment, confirming GLM efficacy and safety in a long-term real-life setting.
